# Ziemssen’s Cyclopœdia

**Published:** 1878-01

**Authors:** 


### Books Reviewed.

*Cyclopædia of the Practice of Medicine.* Edited by Dr. H. VON ZIEMSSEN. Vol. XVI. Diseases of the Locomotive Apparatus and General Anomalies of Nutrition. ALBERT H. BUCK, M. D.,

American Editor. New York: Wm. Wood & Co., 1877. Buffalo: J. H. Matteson. pp. 1060.

This is one of the largest volumes of the series, although the biograpical sketches of the authors have not been published, owing to the fact that they were not received in time from Germany. They will be published in the next volume. The remaining volumes will appear in the following order. Volume XIV. in December, 1877. Volume XIII. in March, 1878. Volume XVII. in June, 1878. Volume VIII. in September, 1878, and the last, Volume IX in December, 1878. These five volumes will probably contain 1000 pages each. The work in the German, with the exception of the volume upon skin diseases, Vol. IX. is practically finished, so that it is absolutely certain that seventeen volumes will complete the work. Thus in a little more than a year will this series be brought to a close. This will be welcome news to many who, under the promise of fifteen volumes of from 500 to 700 pages to be issued at short intervals, were induced to subscribe to a work that has been increased to seventeen volumes of 1000 pages.

Volume XVI contains three articles by Prof. H. Senator, of Berlin. Diseases of the Locomotor Apparatus; translated by E. Buchanan Baxter, M. D. and treating of the Rheumatic affections of the joints and muscles; Gout; Arthritis Deformans, and Rickets. Diabetes Mellitus is translated by Frank P. Foster, M. D., while Diabetes Insipidus is translated by Henry P. Bowditch, M. D. Slight Disorders caused by catching cold, by Prof. E. Seitz, of Geissen, is translated by Dr. Baxter, as is also the next article by Prof. H. Immermann of Basel; General Disorders of Nutrition,—Anæmia, Chlorosis, Progressive Pernicious Anæmia. Corpulence is also presented by Prof. Immermann. Scrofulosis and affections of the Lymphatic Glands in general, by Dr. Birch Hirschfeld, is translated by Godfrey Aigner, M. D.

Prof. H. Immermann, under the treatment of Anæmia recommends transfusion in many cases. He says that a favorable result may be anticipated from a timely resource to transfusion whenever acute oligæmia is brought on by loss of blood in a previous healthy person. Acute anæmia after post-partum hemorrhage offering the best chances of success. The modes of transfusion are considered, and the conclusion arrived at, that defibrinated human blood is by far the best. We have not the space to notice the various articles at length, but would say in brief that as a volume this will rank with any other in the series published. C.

